# Phylogenomics of phosphoinositide lipid kinases: perspectives on the evolution of second messenger signaling and drug discovery

**DOI:** 10.1186/1471-2148-11-4

**Published:** 2011-01-05

**Authors:** James R Brown, Kurt R Auger

**Affiliations:** 1 Computational Biology, Quantitative Sciences, GlaxoSmithKline, 1250 South Collegeville Road, UP1345, P.O. Box 5089, Collegeville PA 19426-0989, USA; 2 Cancer Epigenetics, Oncology R&D, GlaxoSmithKline, 1250 South Collegeville Road, UP1110, P.O. Box 5089, Collegeville PA 19426-0989, USA

## Abstract

**Background:**

Phosphoinositide lipid kinases (PIKs) generate specific phosphorylated variants of phosatidylinositols (PtdIns) that are critical for second messenger signaling and cellular membrane remodeling. Mammals have 19 PIK isoforms spread across three major families: the PtIns 3-kinases (PI3Ks), PtdIns 4-kinases (PI4Ks), and PtdIns-P (PIP) kinases (PIPKs). Other eukaryotes have fewer yet varying PIK complements. PIKs are also an important, emerging class of drug targets for many therapeutic areas including cancer, inflammatory and metabolic diseases and host-pathogen interactions. Here, we report the genomic occurrences and evolutionary relationships or phylogenomics of all three PIK families across major eukaryotic groups and suggest potential ramifications for drug discovery.

**Results:**

Our analyses reveal four core eukaryotic PIKs which are type III PIK4A and PIK4B, and at least one homolog each from PI3K (possibly PIK3C3 as the ancestor) and PIP5K families. We also applied evolutionary analyses to PIK disease ontology and drug discovery. Mutated PIK3CA are known to be oncogenic and several inhibitors are in anti-cancer clinical trials. We found conservation of activating mutations of PIK3CA in paralogous isoforms suggesting specific functional constraints on these residues. By mapping published compound inhibition data (IC50s) onto a phylogeny of PI3Ks, type II PI4Ks and distantly related, MTOR, ATM, ATR and PRKDC kinases, we also show that compound polypharmacology corresponds to kinase evolutionary relationships. Finally, we extended the rationale for drugs targeting PIKs of malarial *Plasmodium falciparum*, and the parasites, *Leishmania *sp. and *Trypanosoma *sp. by identifying those PIKs highly divergent from human homologs.

**Conclusion:**

Our phylogenomic analysis of PIKs provides new insights into the evolution of second messenger signaling. We postulate two waves of PIK diversification, the first in metazoans with a subsequent expansion in cold-blooded vertebrates that was post-emergence of Deutrostomia\Chordata but prior to the appearance of mammals. Reconstruction of the evolutionary relationships among these lipid kinases also adds to our understanding of their roles in various diseases and assists in their development as potential drug targets.

## Background

**Figure 1:**
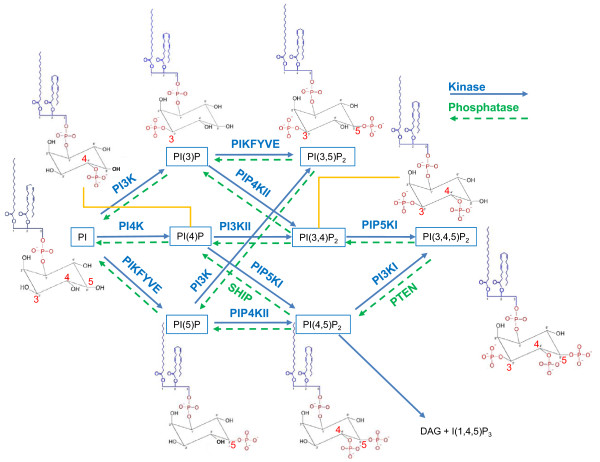
**General pathway for phosphatidylinositide (PI) synthesis**. Major PI types with phosphorylation sites labeled (3,4,5 in red) are shown along with the phophorylation and dephosphorylation reactions catalyzed by different phosphoinositide kinase (PIK) types and phosphatases, respectively. Figure partially adapted from Figure 1 of Weernink *et al*. [6].

Eukaryotic signal transduction is dependent upon various secondary messenger signaling molecules in particular the cellular phospholipids called phosatidylinositols (PtdIns). These phospholipids activate a spectrum of intracellular pathways that regulate multiple core functions including cellular metabolism, cell cycle and survival, protein synthesis, cell polarity and motility, and vesicle trafficking. Phosphorylations around the inositol ring generates various phosphoinositides (PIs) which allow for functional specificity in cell signaling and cellular membrane remodeling [1]. Known PIs include PtdIns-3-phosphate (PtdIns-3-P), PtdIns-4-phosphate (PtdIns-4-P), PtdIns-5-phosphate (PtdIns-5-P), PtdIns-3,4-*bis*phosphate (PtdIns-3,4-P_2_), PtdIns-3,5-*bis*phosphate (PtdIns-3,5-P_2_), PtdIns-4,5-*bis*phosphate (PtdIns-4,5-P_2_), and PtdIns-3,4,5-*tris*phosphate (PtdIns-3,4,5-P_3_). Cellular syntheses of these various PIs are regulated by certain phosphatases and distinct families of lipid-specific kinases called phosphoinositide lipid kinases or PIKs (Figure 1).

PIKs can be broadly categorized into three major protein types: PtIns 3-kinases (PI3Ks), PtdIns 4-kinases (PI4Ks), and PtdIns-P (PIP) kinases (PIP5Ks and PIP4Ks). PI3Ks has eight known isoforms in mammals which are differentiated into three to four classes by amino acid sequence homology, regulator domains and activation cascades [2,3]. Mammals have three Class Ia PI3Ks sharing homologous catalytic polypeptide subunits p110α (PIK3CA), p110β (PIK3CB) and p110δ (PIK3CB). (For clarity, HUGO gene names are in parenthesis and used throughout to refer to their protein products.) Class Ia PI3Ks are heterodimeric protein complexes consisting of the catalytic and regulatory subunits. Class Ia kinases are multi-domain proteins having, in order from the N-terminus, a binding domain for p85 regulatory proteins, a RAS binding domain (RBD) as well as C2, helical (PIK) and catalytic domains. The sole Class Ib PI3K, PIK3CG, has a distinctive p101 regulatory domain as well as homologous, RBD, C2, helical and p110γ catalytic domains. Class II PI3Ks lack either p85 or p101 regulatory domains yet have a p110 catalytic domain and the other domains. In humans, there are three Class II isoforms, PIK3C2α (PIK3C2A), PIK3C2β (PIK3C2B) and PIK3C2γ (PIK3CG). Class III PI3K is represented by a single isoform, PIK3C3 (also known from yeast studies as vacuolar protein-sorting defective 34 or Vps34), and is the least complex PI3K kinases having only C2, helical and catalytic domains.

There are two types of PI4Ks each comprised of two known paralogous isoforms in vertebrates. These are known as Type II, PI4KIIα (PI4K2A) and PI4KIIβ (PI4K2B), and Type III PI4KIIIα (PIK4CA) and PI4KIIIβ (PIK4CB) [4,5]. PI4KIIIs share sequence homology with PI3K and together comprise the largest family of phosphoinositide lipid kinases. PI4KII isoforms do not share sequence homology with either PI4KIII or PI3K kinases. The third PIK family is the phosphatidylinositol-4-phosphate 5-kinases (PIP5K) or PIP kinases which lack primary sequence or structural homology to either PI3Ks or any type of PI4Ks [6]. In mammals, there are three types of PIP kinases or PIPKs. Type I PIP5K occurs as three homologs known as PIP5K1-α (PIP5K1A), PIP5K1-β (PIP5K1B) and PIP5K1-γ (PIP5K1C). Type II phosphatidylinositol-4-phosphate 5-kinase also has three isoforms called PIP4K2-α (PIP4K2A), PIP4K2-β (PIP4K2B) and PIP4K2-γ (PIP4K2C). Both Type I and II PIPKs are homologs to yeast Mss4p. A third PIPK is PIKFYVE has been recently designated as PIP5K3 and includes yeast Fab1p.

PI3K-AKT-mTOR signaling is a central regulatory axis for many key cellular functions including cell cycle, protein synthesis and glucose metabolism. Thus it is not surprising that many diverse disease etiologies have been associated with dysfunctional PIKs including cancer, diabetes and heart disease [7]. Phosphatidylinositol 3-kinase α peptide (PIK3CA) is highly mutated in colon, brain and gastric cancers where apparent gain-of-function mutations confer increased activity for this lipid kinase [8,9]. Several small molecule inhibitors of PIK3CA are in anti-cancer clinical trials [10,11]. The paralog PIK3C-γ or PIK3CG is broadly implicated in many diseases due to its role as a downstream signaling component of chemokine receptors that modulate inflammatory pathways [12]. For example, PIK3CG has been suggested as a heart disease target because of its roles in atherosclerosis related inflammation [13] and pathogenesis of cardiac hypertrophy and heart failure [14]. Recent studies also suggest that PI4KIIIs are essential host factors utilized by the hepatitis C virus (HCV) to remodel the intracellular matrix for virus replication and release [15,16].

PtdIns and PIKs are evolutionary well conserved. In fact, inositol precursors are found in bacteria and archaea although phosphorylated derivatives are strictly eukaryotic [17]. Homologs for all three PIKs groups (PI3K, PI4K, and PIPK) occur across major taxonomic clusters of eukaryotes although the number of isoforms is highly variable with greater complexity among metazoans, especially the vertebrates [18]. Clearly, the emergence and diversification of PIKs second messenger signaling is a hallmark, and potential facilitator, of eukaryotic evolution. However, questions remain about the antiquity of the various PIK subfamilies and the timing of the radiation and divergence of these lipid kinases relative to the emergence of major taxonomic groups. This knowledge is potentially relevant to biomedical interests in PIKs such as in the interpretation and design of model organism studies, characterization of mutations and polymorphic variants and evaluation of potential polypharmacology [19]. In addition, kinase inhibitors have been suggested as novel therapeutics against devastating eukaryotic parasitic diseases such as malaria, caused by the Apicomplexa protists, *Plasmodium falciparum *and *P. vivax *[20], as well as leishmaniasis and trypanosomiasis that result from infections of *Leishmania *sp., and *Trypanosoma *sp., respectively, both members of the Kinetoplastids [21]. Here, we provide a comprehensive genomic survey and phylogenetic analyses of the major types of PIKs. We show the significance of these results in understanding the evolution of second messenger signaling as well as exploiting PIKs as drug targets for various therapeutic areas.

## Results

### Phylogeny of PI3K and PI4KIII kinases

In this study, we focused on the phylogenetic analyses of the phosphoinositide lipid kinases from representative species across seven major taxonomic groups: the Mammalia, Vertebrata (specifically cold-blooded vertebrates), Chordata/Deutrostomia, Protosomatia (insects/arthropods), Pseudocoelomata (nematoda worms), Viridiplantae (green plants), Fungi as well as various protists with an emphasis on human eukaryotic parasitic groups Apicomplexa and Kinetoplastids. We retrieved amino acid sequences from GenBank NonRedundant (nr) and species-specific databases via BLASTP [22] searches using human PIK proteins as the initial queries (see Methods). As necessary, sequences from other species were used as queries to obtain further homologs. While all homologs were initially collected, representative species for each taxonomic major group were selected for phylogenetic analyses on the basis of sequence completeness which was not consistent across all genomes sequences. For example, mammalian kinases could be consistently represented by human (*Homo sapiens*), rat (*Rattus norvegicus*) and mouse (*Mus musculus*) sequences while Chordata/Deutrostomia were represented by an aggregate of one or more homologs from the genome sequences available for species of Deutrostomia (*Strongylocentrotus purpuratus*, sea urchin), Tunicata (*Ciona intestinalis*, tunicate) and/or Cephalochordata (*Branchiostoma floridae*, Florida lancelet or amphioxus). For each of the three major PIKs families, sequences were aligned, edited to the core conserved amino acids and phylogenetic analyses were performed.

**Figure 2:**
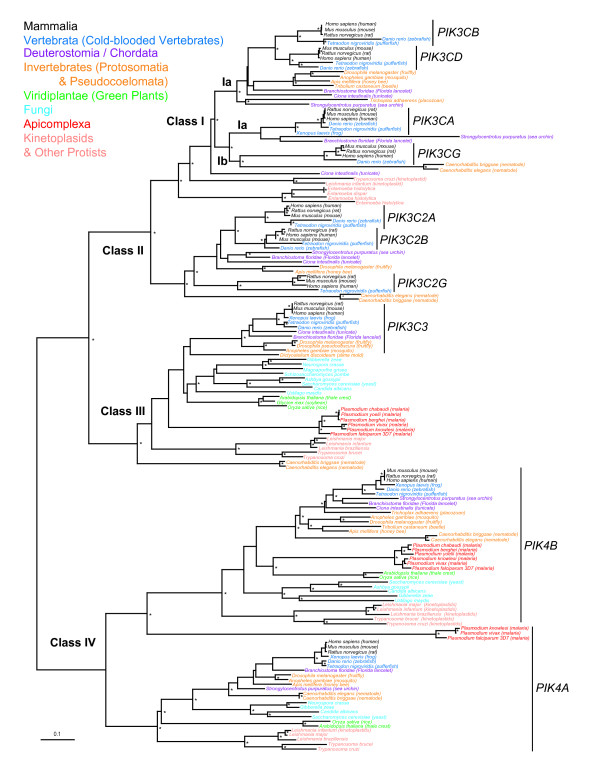
**Phylogenetic tree of phosphoinositide 3-kinases (PI3K) and Type III phosphoinositide 4-kinases (PI4K)**. PI3K proteins are labeled according to HUGO human gene name with classes identified by commonly accepted nomenclature [2]. Main organism taxonomic groups are color coded. For simplicity, Protosomatia (insects/arthropods), Pseudocoelomata (nematode worms) are considered collectively as Invertebrates. The tree was reconstructed by neighbor-joining (NJ) method using protein distance matrices of core conserved amino acids (see Methods). Asterisks ("*") indicate those nodes supported 70% or greater in 1000 bootstrap replicate NJ trees and 0.95 Bayesian posterior probability. Scale bar represents 0.1 expected amino acid residue substitutions per site.

PI3K and PI4K type III (PI4KIII) kinases form the largest homologous group which collectively comprise of 10 distinct isoforms in vertebrates. Phylogenetic analysis shows a distinct clustering of the different subfamilies with high bootstrap and Bayesian posterior probability for most clades (Figure 2). PI4KA and PI4KB (phosphatidylinositol 4-kinase catalytic α and ß, respectively) are significantly diverged from each other as well as PI3K subfamily proteins. Both PI4KA and PI4KB are represented throughout the major taxonomic groups as single genes (Additional files 1 and 2).

The eight isoforms of the PI3K family have a specific hierarchy with PIK3C3 (phosphoinositide-3-kinase, class III) the most divergent relative to PIK3C2 (phosphoinositide-3-kinase, class II) and PIK3C (phosphoinositide-3-kinase, class I) proteins. PIK3C3 occurs in all major taxonomic groups and is the sole PI3K class protein encoded in the genomes of plants, fungi and Apicomplexan (*Plasmodium *sp.). However, Kinetoplastids species have a second PI3K which appears intermediate in homology to Class I and II kinases of metazoans.

PIK Classes Ia, Ib and II cluster together with high bootstrap/probability values and, within each class, the nodes clustering the various isoforms also have strong bootstrap and Bayesian posterior probability support. Class II kinases are specific to metazoans. Of the three isoforms, PIK3C2G (phosphoinositide-3-kinase, class 2, γ polypeptide) is the most divergent and appears common to all species. Nematodes and arthropods are not monophyletic, which is likely an artifact of tree reconstruction resulting from too few phylogenetically informative sites to resolve the evolutionary relationships among these invertebrate species. PIK3C2A (α polypeptide) and PIK3C2B (β polypepetide) are both vertebrate specific and form a strongly supported clade. Deutrostomia/Chordata species have a single PIK3C2 ortholog which, although not entirely well resolved in the overall PI3KC2 cluster, appears to be ancestral to both PIK3C2A and PIK3C2B suggesting the latter two proteins evolved from a gene duplication event in early cold-blooded vertebrates.

Similarly, Class I PI3Ks are mainly found in metazoans with a single isoform in invertebrates. Phylogenetic analysis shows two distinct subclusters of duplicated vertebrate sequences, one of PIK3CB and PIK3CD while the other is comprised of PIK3CA and PIK3CG. Each subcluster has a single ancestral ortholog gene which is found in all three species representing the Deutrostomia/Chordata. Therefore, at least two further gene duplications occurred after the emergence of true vertebrates, one lead to PIK3CB and PIK3CD while the other resulted in PIK3CA and PIK3CG.

### Phylogeny of Class II PI4K kinases

**Figure 3:**
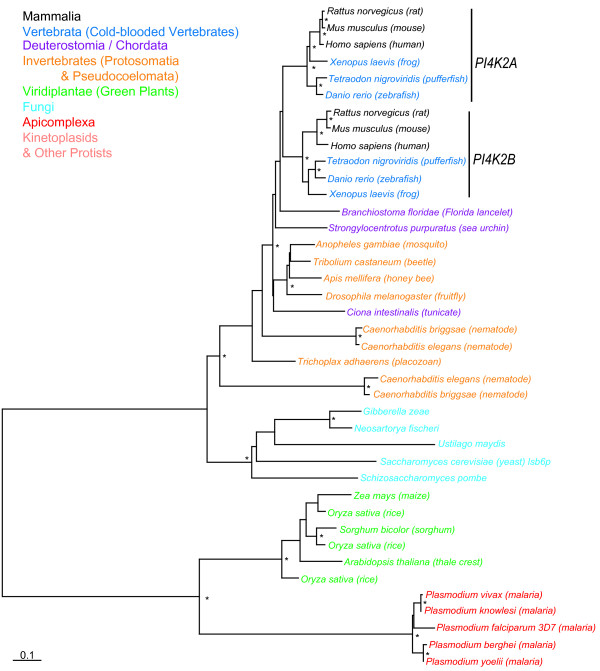
**Phylogenetic tree of Type II phosphoinositide 4-kinasess kinases (PI4K2)**. Nomenclature and phylogenetic reconstruction methods are as described for Figure 2.

Type II PI4K kinases (PI4KII) do not share amino acid sequence homology with either PI4KIII or PI3K kinases (Additional files 1 and 3). Mammalians and other vertebrates have two isoforms, PI4K2A and PI4K2B (phosphatidylinositol 4-kinase type 2 α and β, respectively) which appear to have evolved from an early vertebrate gene duplication as similarly seen for PI3K subfamilies (Figure 3). BLASTP database searches revealed single copy PI4K homologs in all other species except plants having multiple isoforms while Kinetoplastids and other protists appear to be missing this protein. Fungal PI4KII are basal to metazoan isoforms while plant and Apicomplexa PI4KII appear more closely related (although the tree is unrooted). Both phylogenetic relationships are consistent with current views on the fungal origin of metazoans [23] and the role of secondary acquisition of a plastid-like endosymbiont by a single-cell eukaryote during the evolution of the Apicomplexa [24].

### Phylogeny of PIP5 kinases

The PIP5K family consists of three distantly related groups of proteins, PIP5K1 (phosphatidylinositol-4-phosphate 5-kinase, type I), PIP4K2 (phosphatidylinositol-5-phosphate 4-kinase, type II) and PIP5K3 (phosphatidylinositol-4-phosphate 5-kinase, type III) or PIKFYVE. In contrast to PI3K/PI4KIII and PI4KII kinases, the relative evolutionary relationships between these major subfamilies are poorly resolved. Although these proteins share recognizable sequence similarity, in comparison to PI3K/PI4KIII and PI4KII, fewer amino acids could be confidently aligned thus reducing the phylogenetic signal. Furthermore, most eukaryotes have multiple proteins with FYVE finger motifs that are not active kinases but rather kinase substrates [25]. Therefore, we removed from our analysis any proteins with low kinase domain homology (Additional files 1 and 4).

**Figure 4:**
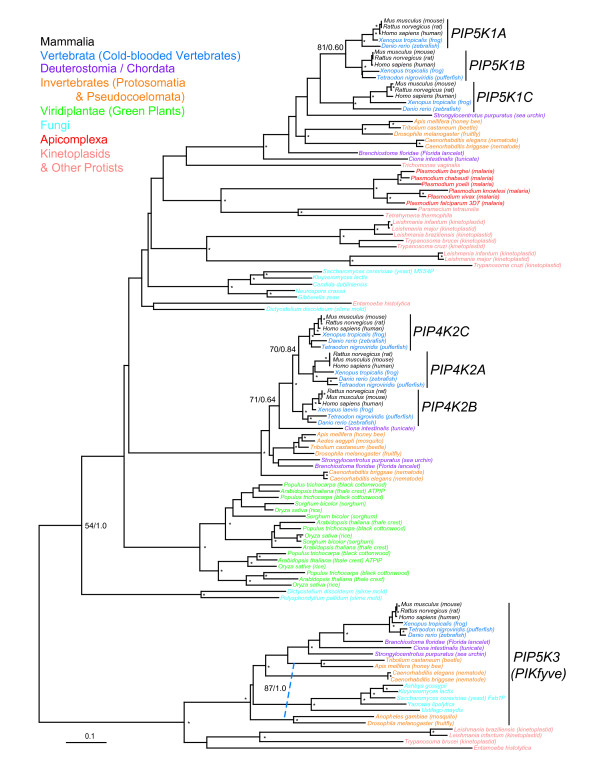
**Phylogenetic tree of phosphatidylinositol-4-phosphate 5-kinases (PIP5K)**. Nomenclature and phylogenetic reconstruction methods are as described for Figure 2. Numbers at major nodes show the percent occurrence of nodes in greater than 50% of 1000 bootstrap replicate neighbor joining trees followed by Bayesian posterior probability values. Dashed blue line shows the placement of *Anopheles gambiae *and *Drosophila melanogaster *in consensus bootstrap neighbor-joining and Bayesian phylogenetic trees which depict invertebrates as monophyletic and ancestral to the Chordata/Deutrostomia/Vertebrata clade.

Nonetheless, all metazoan PIP5Ks were resolved into one of the three subfamilies (Figure 4). All invertebrate species as well as Deutrostomia/Chordata had a single copy of PIP5K1, PIP4K2 and PIP5K3. For PIP5K1, the three known human isoforms had orthologs in cold-blooded vertebrates, again suggesting early vertebrate specific gene duplication events. Moreover, PIP5K1A (α peptide) and PIP5K1B (β peptide) appear to be more similar to each other than either to PIP5K1C (γ peptide) thus might be the result of a more recent divergence event. Similarly, all three vertebrate PIP4K2 proteins were monophyletic with PIP4K2C (γ peptide) and PIP4K2A (α peptide) as sister groups relative to PIP4K2B (β peptide). PIP5K3 is a single copy isoform in all metazoans as well as fungi where the yeast prototype is known as Fab1p. Invertebrates are depicted as paraphyletic and outside of the metazoan cluster in Figure 4. However, both the bootstrap neighbor-joining consensus and Bayesian trees supported the monophyly of metazoan PIP5K3. Fungi also have a second PIP5 kinase, the yeast ortholog Mss4p, which is more similar to PIP5K1.

Plants have multiple, duplicated enzymes which are most similar to PIP5K1 and PIP4K2 kinases although phylogenetic analyses could not confidently cluster plant kinases within either subtype. No PIP5K3 homologs were found in plants. Kinetoplastid species have lineage specific duplications of PIP5K1-like kinases as well as a single PIP5K3 kinase. In contrast, Apicomplexa have a single isoform that is most similar to PIP5K1 kinases. The major nodes connecting plant, yeast, metazoan, Kinetoplastid and Apicomplexa isoforms were not well supported by bootstrap or probability values, therefore no inference can be made about the relative order of evolutionary emergence of the major PIPK types.

## Discussion

### Evolution of Second Messenger Signaling

**Figure 5:**
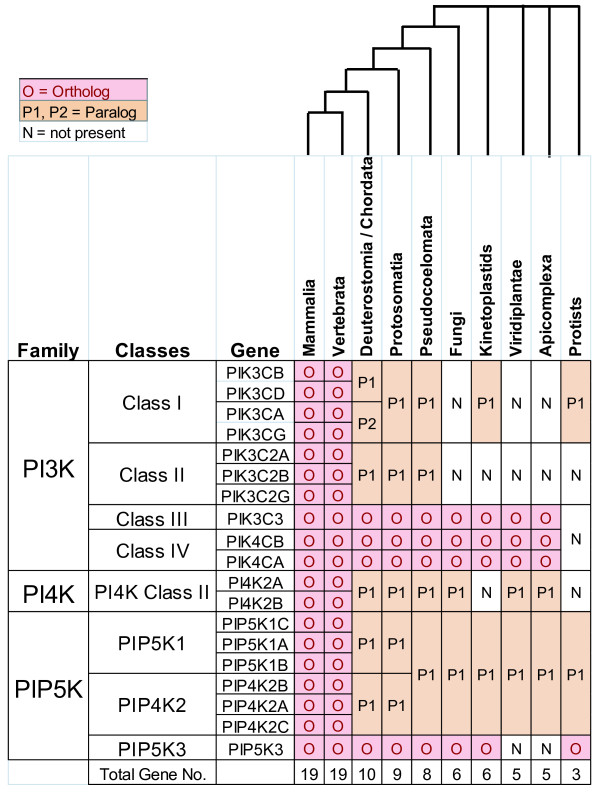
**Phyletic distribution of phosphoinositide kinases**. Shown is a summary of PIK orthologs and paralogs (relative to mammals) across major eukaryotic groups. Cladogram at the top represents relative evolutionary relationships among these groups according to the Tree of Life web project [46].

As central components of second messenger signaling, the evolutionary relationships of PIKs are indicative of the emergence and divergence of this critical signaling pathway in eukaryotes. Integration of multiple PIK phylogenies across major eukaryotic taxonomic groups permits an overview of the evolutionary stages of second messenger signaling from unicellular protists to mammals (Figure 5). Based on their universal conservation across eukaryotes, there is a core group of four PIKs which are type III PIK4A and PIK4B, and at least one homolog from PI3K (possibly PIK3C3) and PIP5K families. PI4KII while present in metazoans, plants, fungi and *Plasmodium *sp., is apparently absent from Kinetoplastids and further searches failed to find significant homologs in the genomes of other protists such as *Trichomonas vaginalis*, *Giardia lamblia *and *Entamoeba histolytica*. *Plasmodium *sp. belongs to the Apicomplexa which are believed to have evolved from the secondary endosymbiosis of a plastid-bearing protist by a primordial eukaryotic cell [24]. Significant evidence supporting this hypothesis are that all Apicomplexa have a non-photosynthetic plastid organelle called the apicoplast and many genes with plant-like origins are integrated into their primary genome [26]. Therefore the occurrence of PI4KII in *Plasmodium *sp. might also be due to the secondary acquisition from a plastid-bearing eukaryotic lineage. Another possible core PIK candidate is PIP5K3 or PIKFYVE which is found throughout metazoans, fungi and some Kinetoplastids. However, we could not find in plants any true PIP5K3 homologs (proteins with a PIP5K kinase domain and FYVE motif) that could be included confidently included our phylogenetic analyses [25].

Among the eight known PI3K family proteins, PIK3C3 is the most likely candidate ancestral kinase of this subfamily. Two pieces of evidence in our analysis support this hypothesis. First, single PIK3C3 orthologs are found in all eukaryotic groups [2]. Second, phylogenetic analysis firmly establishes the early divergence of PIK3C3 from Class I and II PI3Ks. This assessment is congruent with the early pathway catalytic function of PIK3C3 in the generation of PtdIns-3-phosphate (PtdIns-3-P) from a PtdIns precursor. In addition, PIK3C3 has the simplest protein structure among all PI3K kinases insofar as it lacks the RAS binding domains of Class Ia, Ib and II PI3Ks as well as the p85/p110 regulatory domains of Class Ia PI3Ks. Although only the core PIK and catalytic domains were used for the construction of the PI3/4K phylogenetic tree, the branching order of the three PI3K Classes corresponds well with the general trend towards increasing kinase complexity in terms of the number of regulatory domains and the capability to phosphorylate more complex PtdIns derivatives.

With the exception of some Kinetoplastid sequences, Class I and II PI3Ks are metazoan specific proteins. Our phylogenomic analysis suggests an evolutionary trend where increasing organism complexity is associated with two major waves of PIK diversification (Figure 5). First, in early metazoans gene duplications lead to the emergence of Class I and II PIKs as well as PIP5K1 and PIP4K2 families. This is evident from the wider repertoire of PIK isoforms in invertebrates relative to plants, fungi and protists. The second gene expansion occurred in early, true vertebrates. For most PIKs, a series of gene duplications occurred in cold-blooded vertebrates after their divergence from early urochordates but before the emergence of mammals. Representative species of the Deutrostomia (*Strongylocentrotus purpuratus*), Tunicata (*Ciona intestinalis*) and Cephalochordata (*Branchiostoma floridae*) mostly have the same PIK complement as arthropods and other invertebrates. Possible exceptions are the Class Ia and Class Ib PIKs where a single corresponding ortholog for each subclass can be found in the Deutrostomia/Chordata species. Thus the initial diversification of Class I PI3Ks likely occurred in the early deutrostome lineage followed by two additional gene duplications in vertebrates resulting in contemporary complement of four Class I PIKs throughout cold-blooded vertebrates and mammals.

Many different genes spanning wide functional categories have undergone major gene duplications in vertebrates and mammals. However, given the crucial role of second messenger signaling, it is tempting to speculate that increasing multi-cellular complexity was accompanied and, perhaps facilitated, by the evolution of more diverse and specialized PIKs signaling cascades.

### Evolutionary Context for Cancer Mutations

The gene for phosphoinositide-3-kinase α peptide (PIK3CA) is highly mutated in colon, brain and gastric cancers where apparent gain-of-function mutations confer increased activity for this lipid kinase [8,9]. Our phylogenetic analysis shows that PIK3CA is most closely related to other class Ia and Ib kinases, PI3KC-ß (PIK3CB), PI3KC-δ (PIK3CD), and PI3KC-γ (PIK3CG) (Figure 2). Thus further knowledge about tumor-related mutations in PIK3CA might be gained by comparing those variants to orthologs in other species as well as paralogs of related kinases.

**Figure 6:**
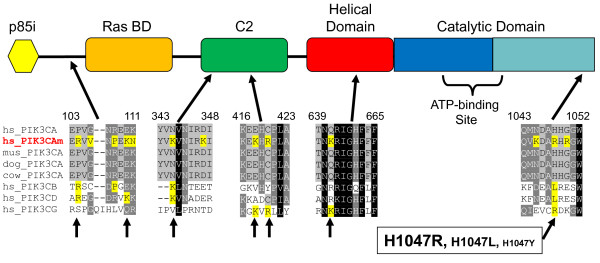
**Occurrence of missense cancer mutations in PIK3CA gene relative to orthologous and paralogous PI3K kinases**. PIK3CA sequences are from human (hs_PIK3CA), mouse (mus_PIK3CA), and dog (dog_PIK3CA) and cow (cow_PIK3CA) as well as the human paralogs PIK3CB (hs_PIK3CB), PIK3CD (hs_PIK3CD), and PIK3CG (hs_PIK3CG). Also included is a composite cancer mutant human PI3KCA (hs_PI3KCAm in red) with amino acid substitutions (mutations) mapped as reported by Samuels *et al*. [8] and the Sanger COSMIC database [27]. Regions of the alignments are shown where a cancer missense mutation is identical to an amino acid occurring in normal human paralogs. Numbers indicate coordinates in normal human PI3KCA. Arrows at the bottom of the alignment point to those specific changes across paralogues. Note for H1047, three different amino acid substitutions have been observed and font size of label indicates the relative high (large font) to low (small font) oncogenic potency of each type [28]. Structural domains were taken from the alignment of PI3K kinases to the PI3K C-γ structure reported by Walker *et al*. [47] and are not drawn to scale.

Alignment of a consensus sequence of non-synonymous cancer mutations reported in the COSMIC database [27] with normal human PIK3CA as well as orthologs from mammals and human paralogues for PIK3CB, PIK3CD and PIK3CG are shown in Figure 6. Several mutations occur in regions of PIK3CA which are conserved throughout mammalian isoforms. At least seven mutations, while non-conserved among PIK3CA orthologs, are conservative changes matching residues in one or more of the three other corresponding human PIK3C paralogs. According to the COSMIC database, one of the most frequent variants observed in cancer is H1047R in the terminal end of the kinase domain which has been shown to be an activating or gain-of-function mutation. The variants H1047L and more rarely, H0147Y, have also been recovered from clinical tumor samples.

Gymnopoulos *et al*. [28] measured the oncogenic potential of the 15 most common PIK3CA mutations found in tumors by introducing retroviral expression vectors with each of the variants into avian cells and measuring their individual efficiencies for tumorigenic transformation. Their functional assays confirmed that the mutation H1047R strongly conferred oncogenic potency while moderate and weak potency was induced by the variants H1047L and H1047Y, respectively. Interestingly, PIK3CA H1047R mutation aligns with an arginine found in normal human PIK3CG while H1047L corresponds to leucine in wild-type PIK3CB and PIK3CD (Figure 6). H1047Y is rarely found in tumors, appears to convey much weaker oncogenic potency than the other two mutations and is not conserved in any Class I PI3K paralog. The correspondence of certain cancer mutations in PIK3CA to those found in normal paralogs suggests that selection pressures in the tumor might limit the range of acceptable amino acid changes. One might speculate that these constrained amino acid changes in a mutated PIK3CA protein could lead to convergence in function, protein interactions or regulation with those of PIK3CB, PIK3CG or PIK3CD. Gymnopoulos and co-workers mapped H1047R/L mutations onto a structural model of PIK3CA which suggested that these changes are located near the hinge region of the activation loop and could serve to increase catalytic activity [28]. Given the importance of mutated kinases in tumor cell viability and their increased exploitation as cancer drug targets, better insights into delineating between passenger and driver mutations might be gained through broader sequence comparisons across different species as well as related protein family members [29,30].

### PIK Inhibitor Polypharmacology

Knight *et al*. reported inhibition data for compounds screened across the entire PIK3C family as well as several more distantly related PI3K-like kinases such as ATM (ataxia telangiectasia mutated), ATR (ataxia telangiectasia and Rad3 related), PRKDC (protein kinase, DNA-activated) and MTOR (mechanistic target of rapamycin) [31]. Their study did not include a phylogenetic or comparative sequence analyses but rather used separate principal component analysis (PCA) plots to compare the statistical space of target similarity versus compound-target inhibition values. More recently, the same group published in Apsel *et al*. the results of several dual inhibitors of both tyrosine and PI3K and PIK4A kinases [32]. Compounds were assayed for inhibition against all Class Ia and Ib PI3Ks, MTOR, PRKDC and PIK4A as well as several tyrosine kinases although not all screening panels had a complete kinase set.

**Figure 7:**
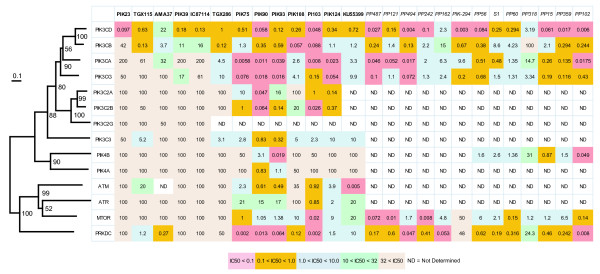
**Phylogenetic tree of PIK3/4III and related protein kinases with IC_50 _values from tested inhibitors**. Compound names in column are as reported by Knight *et al*. [31] (bold font) and Apsel *et al. *[32] (italics). Kinase assay are aligned with their branching order in the phylogenetic tree. IC_50 _values (μM) are shaded according to potency with smaller values being more effective inhibitors of kinase activity. The tree was constructed using the neighbor-joining method as described for Figure 2 with numbers at nodes showing percent support in 1000 bootstrap replications.

Studying and predicting the target spectrum and specificity of any drug is important for determining potential polypharmacology (the modulation of multiple targets by a single compound) as well as evaluating possible drug toxicities (due to undesirable off-target binding). In these respects, we were interested in melding phylogenetic analyses of lipid kinases with compound activity data to as a potential approach to complement chemogenomics studies. We aligned the core kinase domains of PI3Ks, PI4KIIIs, ATM, ATR, MTOR and PRKDC (133 amino acids) and reconstructed a phylogenetic tree (Additional files 1 and 5). Selected compound inhibition data from both studies were mapped to the terminal branches of the respective kinase in the phylogenetic tree (Figure 7). PI3KCD is the most common, potently inhibited kinase in both studies and several compounds shown high specificity such as the compound PIK23 from Knight and co-workers [31]. Other compounds, such as PIK90 and PI103 from Knight *et al*. [31] or PP102 and PP494 of Apsel *et al*. [32], with high potencies across multiple PI3Ks also inhibit one or more distantly related kinases such as ATM, ATR, PRKDC, or MTOR. Interestingly, several compounds show higher potency against the kinase pair PIK3CA and PIK3CG versus PIK3CD and PIK3CB. PIK3CA, PIK3CG and PIK3CB are considered as Class Ia while PIK3CG is classified as Class Ib. However, the compound inhibition profiles generally correlate with the closer evolutionary relationships between PIK3CA and PIK3CG kinases as shown in our trees (Figure 2 and 7).

The published compound data has some limitations insofar that not all possible lipid kinase homologs were assayed. For example, Apsel *et al*. [32] do not report any compound assay data for PIK3C2 or PIK3C3 kinases. However, there is an overall trend for compounds with broad Class I PI3K spectrum to inhibit other PIKs as well as more distantly related kinases. Conversely, compounds with high potency to a subset of Class I PI3Ks tend to be more specific. Selective polypharmacology of PIKs might be an important goal for certain disease indications. Foukas *et al*. suggest that Class Ia PI3Ks have functional redundancy in cancer cells, so inhibition of multiple isoforms might be desirable [33]. Conversely, selective inhibition of a particular PI4KIII isoform might be necessary for developing a well-tolerated anti-hepatitis C viral therapy [15,16]. Further molecular modeling and testing with additional compound chemotypes might help illuminate the particular binding interactions that are involved in compound specificities.

### PIKs as Anti-parasite Drug Targets

Some of the world's most important infectious diseases are caused by eukaryotic single-cell parasites. For example, approximately 243 million cases of malaria and 863,000 attributed deaths were reported globally in 2009 [34]. The causative agents of malaria are the Apicomplexa parasites of which *Plasmodmium falciparum *followed by *P. vivax *are the two most important infectious agents. Globally, millions are afflicted by the diseases Leishmaniasis (kala-azar disease) and trypanosomiasis (Chagas disease and sleeping sickness) which result from infections of two genera of Kinetoplastids, *Leishmania *sp., and *Trypanosoma *sp., respectively [35]. New drugs against these parasites are urgently required because of toxicities of existing therapies, the lack of pediatric formulations and the emergence of drug-resistant strains. The need for novel drugs is particularly acute for malaria where there have been increasing reports of *P. falciparum *strains with reduced clinical response to the latest class of artemisinin-containing drug combinations [36,37].

**Figure 8:**
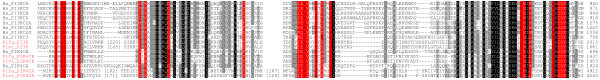
**Malarial and human PIK3/4III kinase domains**. Alignment of kinase domains of PI3K and PI4KIII kinases from *Homo sapiens *(Hs), *Plasmodium falciparum *(Plfa) and *P. vivax *(Plvi). Plasmodium species names and residues implicated in the kinase ATP-binding pocket are highlighted in red. Domains are from the PI3K C-γ (PIK3CG) structure reported by Walker *et al*. [47].

Several previous studies have suggested that kinases are novel target opportunities for these pathogens because of their essential roles in metabolism, signaling and pathogenesis [20,21]. In several ways, our study extends that rationale. The lower complement of PIKs in unicellular eukaryotes relative to metazoans suggests poor pathway redundancy and enhanced probability of essential function to the parasite. A recent screen of a large pharmaceutical compound collection revealed thousands of potential leads including compounds known to inhibit human PI3Ks [38]. The anti-plasmodial activity of these compounds might be through direct inhibition of *Plasmodium *PI3K, although blocking the human host target cannot be discounted because the screens were performed on *P. falciparum *cultured in blood erythrocytes. A comparison of human and *Plasmodium *kinases of the PI3K and PI4KIII types show overall conservation of key catalytic amino acids which suggests that screening human PIK3 compound collections might find further inhibitors of malarial kinases (Figure 8).

As shown in our phylogenetic trees, parasite kinases are divergent from human host homologs suggesting potential scope for developing compounds with specificity to bind *Leishmania*, *Trypanosoma *or *Plasmodium *PIKs. However, Kinetoplastids and Apicomplexa are evolutionary distinct groups. The Apicomplexa, including *Plasmodium *sp., have been proposed to evolve from an ancestor that acquired a secondary plastid through endosymbiosis with a photosynthetic alga [24]. In contrast, comparative genomic analyses of *Trypanosoma cruzi*, *T. brucei *and *Leishmania major *could not find significant evidence supporting similar plastid invasion into those genomes [39]. Our findings of Type II PIK4 kinases being present in plants and Apicomplexa yet absent in Kinetoplastids, additional PI3K genes in Kinetoplastids and the overall non-clustering of these groups in our phylogenetic trees supports the varied evolutionary history of these protists. As such, differential development of kinase inhibitors for the treatment of malaria, leishmaniasis and trypanosomiasis might need to be considered. Malarial genes of putative plastid origins have been suggested to be potential drug targets because of their divergence from human homologs [40]. In our analysis, the Type III PIK4CB and Type II PIK4B proteins of *Plasmodium *sp. cluster with plant orthologs with significant posterior probabilities and high bootstrap values suggesting a plant or cyanobacteria origin for those kinases. Understanding the evolutionary relationships of *Trypanosoma*, *Leishmania *and *Plasmodium *PIKs relative to each other and their human host will further facilitate both drug target selection and the design of counter-screen assays.

## Conclusion

Phylogenomic analyses of PIKs show the progressive complexity of second messenger signaling from unicellular eukaryotes through metazoans and, finally, vertebrates. Furthermore, an evolutionary perspective is a potentially useful framework for characterizing PIKs as drug targets in cancer and parasitic diseases as well as providing insights into the polypharmacology of this important group of kinases.

## Methods

We reconstructed the evolutionary relationships of three major lipid kinase families, PI3K/PI4KIII, PI4KI and PIP kinases, from representative species across seven major taxonomic groups Mammalia, Vertebrata (specifically cold-blooded vertebrates), Deutrostomia/Chordata, Protosomatia (insects/arthropods), Pseudocoelomata (nematoda worms), Viridiplantae (green plants), Fungi (yeasts and other fungi), and human eukaryotic parasitic groups Apicomplexa and Kinetoplastids. Protein (amino acid) sequences were retrieved from GenBank NonRedundant (nr) and species-specific databases via BLASTP [22] (default settings) searches using human PIK proteins as the initial queries. As necessary, sequences from other species or additional paralogs were used to obtain a full set of homologs with a cut-off of E-value ≤ 1.0e^-10^. Initially, large multiple sequence alignments were constructed for all available homologs for each of the three PIK families. Subsequently, representative species were selected on the basis of sequence completeness. For some of the major taxonomic groups, the genome sequence quality was inconsistent, so multiple species were used in each of PIK phylogenetics. For example, mammalian kinases could be consistently represented by human (*Homo sapiens*), rat (*Rattus norvegicus*) and mouse (*Mus musculus*) sequences while Deutrostomia/Chordata were represented by an aggregate of one or more homologs from the genome sequences available for species of Deutrostomia (*Strongylocentrotus purpuratus*, sea urchin), Tunicata (*Ciona intestinalis*, tunicate) and/or Cephalochordata (*Branchiostoma floridae*, Florida lancelet).

Initial multiple sequence alignments were performed using the program CLUSTALW v1.83 [41] with default settings and subsequently, refined manually using the program SEQLAB of the GCG Wisconsin Package v11.0 software package (Accelrys, San Diego, CA, USA). We removed regions with residues that could not be unambiguously aligned or that contained insertions which resulted in edited alignments of the kinase families (length in amino acids or aa) PI3K/PI4KIII (236 aa), PIK4KII (269 aa), PIP (182 aa) and human PIK kinase homologs (133 aa). These multiple sequence alignments used in phylogenetic tree reconstructions in Figure 2, 3, 4 and 7 are included as Additional files 1, 2, 3, 4, 5.

We constructed phylogenetic trees using distance neighbor-joining (NJ) and Bayesian posterior probabilities (BP). NJ trees were based on pair wise distances between amino acid sequences using the programs NEIGHBOR and PROTDIST (Dayhoff option) of the PHYLIP 3.6 package [42]. The programs SEQBOOT and CONSENSE were used to estimate the confidence limits of branching points from 1000 bootstrap replications. BP trees were constructed using the software MrBayes v3.0B4 [43,44]. Bayesian analysis used the mixed model of sequence evolution with random starting trees. Markov chains were run for 10^6 ^generations, burn-in values were set for 10^4 ^generations, and trees sampled every 100 generations. All trees were visualized using the program TREEVIEW v1.6.6 [45].

PIK3CA mutations used to generate the composite cancer mutant human PI3KCA sequence in Figure 6 were obtained from Samuels *et al*. [8] and the Sanger COSMIC database [27]. The IC_50_s shown in Figure 7, were obtained from published and supplementary materials provided by Knight *et al. *[31] and Apsel *et al. *[32]. For the later study, we selected to use those compounds with PI3K inhibition IC_50_s which had been either shown in Figure 2a and/or were in supplementary material with determined PI4KA IC_50_s of 1.0 μM or better potencies.

## Competing interests

The authors declare that they have no competing interests.

## Authors' contributions

JRB conceived the study, carried the bioinformatics analyses, and drafted the manuscript. Both JRB and KRA co-wrote the submitted manuscript. All authors read and approved the final manuscript.

## Supplementary Material

Additional file 1 **Accession numbers of protein sequences used in phylogenetic reconstructions**. NCBI or EMBL accession numbers associated with the operational taxonomic labels (OTUs) for multiple sequence alignments in Additional files 2-5 which were used in phylogenetic reconstructions for Figures 2, 3, 4 and 7. Click here for file

Additional file 2 **Edited multiple sequence alignment of PI34K kinases**. Multiple sequence alignment of PI34K kinases and related sequences used for phylogenetic reconstruction shown in Figure 2. Species, protein names and GenBank accession numbers (as identified by OTU label) are given in Additional file 1. Sequences are in MSF format with first line commenting on file contents. Click here for file

Additional file 3 **Edited multiple sequence alignment of PI4K Type II kinases**. Multiple sequence alignment of PI4K Type II and related sequences used for Figure 3. Species, protein names and GenBank accession numbers (as identified by OTU label) are given in Additional file 1. Sequences are in MSF format with first line commenting on file contents. Click here for file

Additional file 4 **Edited multiple sequence alignment of PIPK kinases**. Multiple sequence alignment of PIPK and related sequences used for Figure 4. Species, protein names and GenBank accession numbers (as identified by OTU label) are given in Additional file 1. Sequences are in MSF format with first line commenting on file contents. Click here for file

Additional file 5 **Edited multiple sequence alignment of human PI34K and related kinases**. Multiple sequence alignment of PI34K and related sequences used for Figure 7. Species, protein names and GenBank accession numbers (as identified by OTU label) are given in Additional file 1. Sequences are in MSF format with first line commenting on file contents. Click here for file
